# Fighting Depression: Action Video Game Play May Reduce Rumination and Increase Subjective and Objective Cognition in Depressed Patients

**DOI:** 10.3389/fpsyg.2018.00129

**Published:** 2018-02-12

**Authors:** Simone Kühn, Fabrice Berna, Thies Lüdtke, Jürgen Gallinat, Steffen Moritz

**Affiliations:** Department of Psychiatry and Psychotherapy, University Medical Center Hamburg-Eppendorf, Hamburg, Germany

**Keywords:** major depression, action video game, randomized control trial, training, rumination

## Abstract

Cognitive deficits are common in depression and may persist following the resolution of affective symptoms. However, therapeutic strategies that successfully target cognitive impairments are lacking. Recent work has demonstrated that playing action video games leads to improvements in cognition, in particular executive function, in healthy individuals. We therefore set out to test whether playing video games can reduce symptoms associated with depression. We focussed on depressive symptoms and on rumination, since rumination is a good predictor of depression and may contribute to triggering depression. We recruited 68 clinically depressed individuals (mean age: 46 years, 47 females) that were randomized into the training group playing a fast paced action video game for 6 weeks or a waitlist control group. Before and after training participants completed online questionnaires and a neuropsychological test battery. Only participants who actually played the game were included in the analysis. The final sample consisted of *n* = 21 training group and *n* = 29 waitlist control group. The training group showed significantly higher subjective cognitive ability, as well as lower self-reported rumination at posttest in contrast to the control group (although these findings do not survive Bonferroni correction). On a subsample with cognitive performance data (*n* = 19) we detected an improvement in executive function (Trail Making Task A and B) in the training compared with the control group. The results show that the fast paced action video game employed in the present study improved Trail Making performance and may reduce rumination and enhance subjective cognitive ability. Future research may focus on the investigation of the precise cognitive profile of effects.

## Introduction

Depression is among the major causes of global disease burden (Vos et al., 2012). In 2010, it was the second leading medical cause of disease burden, with particularly high numbers of disability in working age (Ferrari et al., 2013), making it a particularly costly disease for society. Although affective symptoms, namely low mood accompanied by low self-esteem and a loss of interest in activities that others find enjoyable, are at the core of current diagnostic criteria for depression, cognitive dysfunction also plays a crucial role. Importantly, while treatments such as psychotherapy or medication have proven effective in improving mood, cognitive deficits oftentimes persist (Baune et al., 2010). Objective measures of executive function, attention, and short- and long-term memory reveal cognitive impairments in individuals with major depression (Hammar and Ardal, 2009; Gonda et al., 2015). Additionally, there is a discordance between the objective measures of cognitive dysfunction and the subjectively reported cognitive complaints, the latter being even more pronounced (Moritz et al., 2004), which may be due to a bias toward negative cognitive schemata that is commonly observed in major depression (Svendsen et al., 2012; Lam et al., 2014).

Recent studies investigating the effects of training interventions using video games have shown improvements in cognitive function, although these studies have almost exclusively been performed on healthy young adults. Interestingly, these prior studies consistently show that action video game interventions in particular are associated with specific improvements in attention and processing speed (Green and Bavelier, 2003; Dye et al., 2009) as well as in executive functions such as flexible updating of task-relevant information (Colzato et al., 2013). A recent meta-analysis on the cognitive domains that benefit from commercial video game playing has shown that particularly executive functions (or “information processing” as they call it) show improvements (Powers et al., 2013). Many of these previous studies on video games have investigated expertise effects, that is, they compared habitual gamers with non-gamers cross-sectionally. However by now a considerable number of studies also demonstrate beneficial effects of video game training on cognition in longitudinal designs, supporting a causal relationship.

Previous studies investigating the effects of video games in the context of depression have mostly used specific custom-made games for training that explicitly included elements of cognitive behavioral therapy (CBT) and psychoeducation such as SPARX (Merry et al., 2012; Poppelaars et al., 2016). Other studies have used so-called exergames, which are video games that promote physical exercise. Since regular fitness training is known to improve depression (Kvam et al., 2016), it may not be overly surprising that these exergames likewise lead to an alleviation of symptoms (Li et al., 2016). However, we suspect that compliance with physical exercise regimes is generally lower and more patients will enjoy a video game rather than an exercise intervention. A recent survey conducted in the United States reported that in all age groups, more than 185 million people play and enjoy videogames (≈ 54%; Entertainment-Software-Association, 2017). First evidence for potentially positive effects of commercial video game playing on depression comes from a study that gave patients with major depression the choice between three different commercial puzzle video games (Russoniello et al., 2013). After an instructed 6 h of game play spread over 1 month, the video game group showed significant decreases in depression severity compared with a control group that surfed Internet websites on depression for the same amount of time. However, puzzle-like video games have not been associated as systematically with cognitive benefits encompassing several executive functions as action video games in healthy samples (Green and Bavelier, 2012; Green et al., 2012; Strobach et al., 2012). We therefore set out to test the effects of action video gaming on depressive symptoms and cognitive function in a depressed population.

Based on this prior evidence of improvement in the domain of executive functioning, in response to training with action video games in healthy populations, the present study set out to investigate the effects of action video game training on cognition and depression severity in depressed patients. Furthermore we also assessed symptoms associated with depression such as rumination, since rumination is an important predictor of depressive symptoms (Nolen-Hoeksema, 2000). We hypothesized that depressed patients who complete an action video game intervention would show a larger reduction in depressive symptoms and rumination, and an increase in both objective and subjective measures of cognition, compared with depressed patients in a passive (wait-list) control group.

## Methods

### Participants

Former research participants of the Department of Psychiatry and Psychotherapy, University Medical Center Hamburg-Eppendorf (Germany) with a verified diagnostic status of major depression or dysthymia [based on DSM-IV criteria acquired in the context of the Mini International Neuropsychiatric Interview (MINI; Ackenheil et al., 1999)] within the last 6–12 months, were informed about the study via personalized Email correspondence. All participants had given explicit permission to be contacted for future studies. Inclusion criteria were age between 18 and 65 years, willingness to provide electronic informed consent and to participate in anonymous (internet-based) surveys as well as a diagnosis of major depression. We excluded patients with psychosis, bipolar disorder, and major neurological disorders.

**Figure 1:**
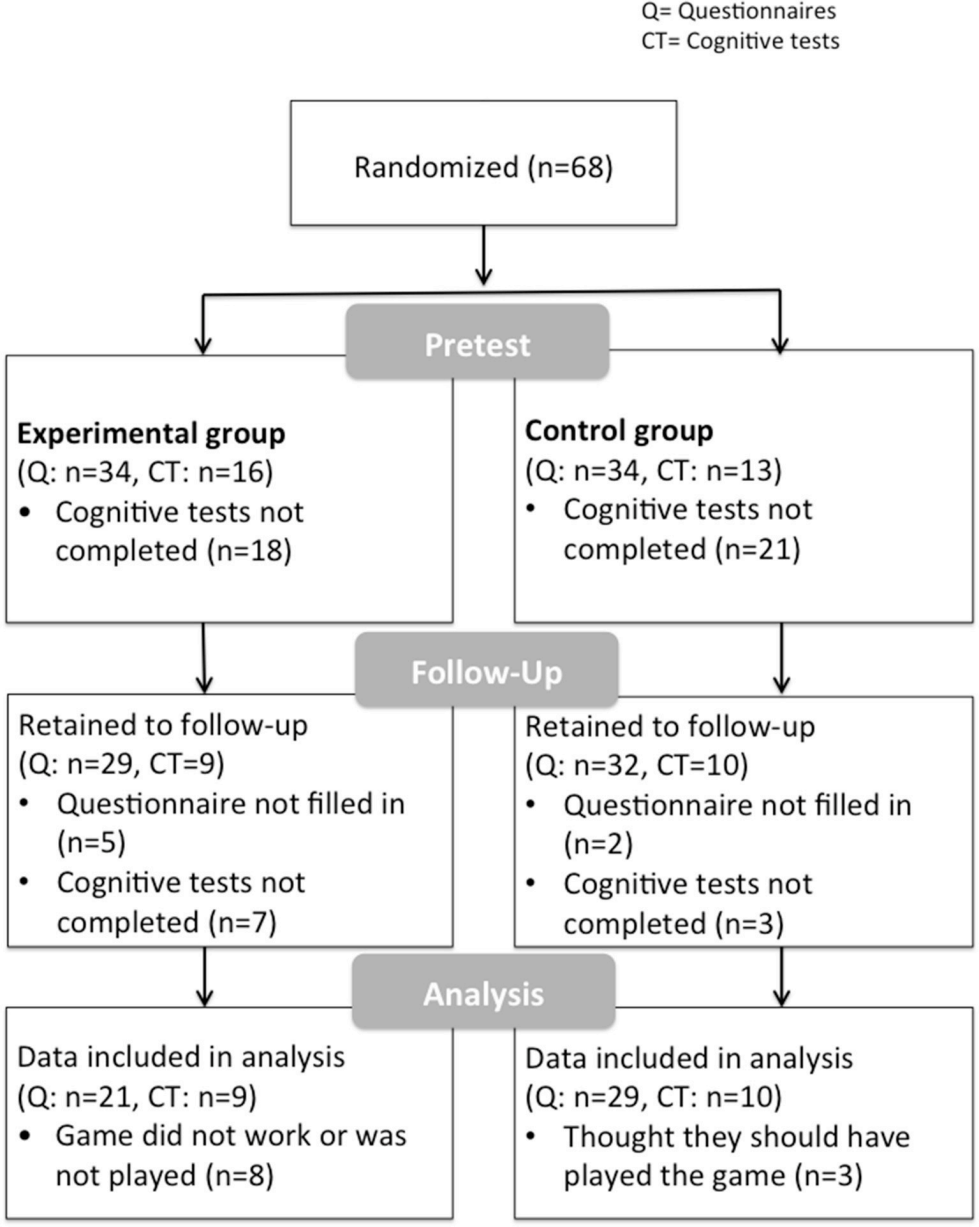
Flowchart illustrating participants' progress through the phases of the randomized controlled trial.

We assigned 68 participants randomly to the experimental or control group (Figure 1). This study was carried out in accordance with the recommendations of the ethics committee of the German Society of Psychology (DGPs). All participants gave written informed consent in accordance with the Declaration of Helsinki. The first Email contained a weblink directing interested parties to the pretest survey. The trial was created using Questback, which does not store IP addresses. Multiple log-ins via the same computer were prevented by means of “cookies”. All participants received a manual on relaxation techniques for their participation but no monetary incentive.

### Procedure

The survey consisted of the following parts: invitation, informed consent (mandatory), demographic section (e.g., gender, age), medical information (e.g., psychiatric diagnoses), assessment of psychopathology (see Questionnaires section below). At the end of the questionnaires, participants received a link directing them to online cognitive tests (Inquisit, Seattle, WA: Millisecond Software; see Cognitive Tests section below). Then, participants in the experimental group were given access to the action video game “Boson X” (see Action Video Game “Boson X” section below) via Email within 24 h. The participants in the control group received access to the game only after the posttest and did therewith not receive a training intervention during the study phase, and therefore represent a passive (wait-list) control group. Six weeks after the pretest assessments, participants were invited to participate in the posttest. Up to two reminders were dispatched in case participants failed to complete the posttest. The posttest consisted of the same questionnaires and cognitive tests as the pre-assessment with some additional questions asking for an evaluation of the video game.

### Questionnaires

#### Patient health questionnaire (PHQ-9, Kroenke et al., 2001)

The PHQ is a self-administered diagnostic instrument for common mental disorders; the PHQ-9 is the depression module scoring each of the nine DSM-IV criteria (from 0 = “not at all” to 3 = “nearly every day”) and refers to the past 2 weeks (e.g., “Over the last 2 weeks, how often have you been bothered by any of the following problems? Little interest or pleasure in doing things”). Cronbach's alpha = 0.86–0.89, test-retest reliability, *r* = 0.84.

#### Beck depression inventory (BDI, Hautzinger et al., 1995)

The BDI measures the severity of depression during the past week, and consists of 21 items, each scored on a scale value from 0 to 3 (e.g., “0 = I do not feel sad, 1 = I feel sad, 2 = I am sad all the time and I can't snap out of it, 3 = I am so sad and unhappy that I can't stand it.”). The test has been shown to have high test-retest reliability (*r* = 0.93; Beck et al., 1996) and a Cronbach's alpha of 0.86 as well as high internal consistency (0.81–0.86; Beck et al., 1988).

#### Response styles questionnaire (RSQ, Treynor et al., 2003)

The RSQ, consists of 10 items each scored on a scale value from 1 = “almost never” to 4 = “almost always” and measures rumination as a trait (e.g., “think about how sad you feel”). It has been shown that this measure is unconfounded with depression. The RSQ has a Cronbach's alpha of 0.85 and test-retest correlation of *r* = 0.62.

#### Subjective scale to investigate cognition in schizophrenia (SSTICS, Stip et al., 2003)

This 21-item self-report questionnaire has been developed to assess subjective cognition in schizophrenia patients covering several cognitive domains namely working memory, episodic memory, semantic memory, attention, executive functioning (consistency of the scale reflected by Cronbach's alpha = 0.86, test-retest reliability across a mean interval of 11 days: *r* = 0.82). When rating their responses, we asked participants to refer to the past 2 weeks (e.g., “Do you have difficulty memorizing things, such as a grocery list or a list of names?”). Although the SSTICS was originally developed for schizophrenia patients, the cognitive domains covered in the questionnaire are not specific to schizophrenia, but are also highly relevant to depression. We therefore decided to use the SSTICS in the present study. Convergent validity of the SSTICS has been shown in terms of an association with the Positive and Negative Symptom Scale (PANSS) cognitive subscale and the Frankfurt-Pamplona Subjective Experiences Scale (FPSES; Lecardeur et al., 2009). Most studies on schizophrenia patients have shown a significant correlation between objective cognitive performance and SSTICS score (Homayoun et al., 2011). We subdivided the total SSTICS score into a subscore for working memory (item 1, 2, Cronbach's alpha = 0.55), episodic memory (item 3–9, Cronbach's alpha = 0.58), semantic memory (item 10, 11, Cronbach's alpha = 0.58), attention (item 12–16, Cronbach's alpha = 0.61) and executive functioning (item 17–19, Cronbach's alpha = 0.67), to test which domain is affected the most (Stip et al., 2003).

#### Habitual video game playing

Habitual video game playing was assessed at pretest by means of two questions: “How many hours do you play video games on a regular weekday?” and “How many hours do you play video games on a regular day of the weekend?” (Kühn and Gallinat, 2014). Based on these responses we calculated the average time spent on video games per participant per week.

#### Assessment of video game play time during the training period

At posttest, participants were asked how frequently they engaged in the training (1 = “never”, 2 = “one time per week or less,” 3 = “two times per week,” 4 = “three times per week,” 5 = “four times per week,” 6 = “five times per week,” 7 = “six times per week,” 8 = “every day of the week”). Participants who were assigned to the training group and indicated that they never played the game were excluded. We also collected logfiles of the game play, however only a small subset of participants (*n* = 13) returned these logfiles via Email, therefore we used the self-report measure to assess game play times during the training period. However the self-report data was highly correlated with the play time extracted from the logfiles [*r*_(13)_ = 0.842, *p* < 0.001].

### Cognitive tests

#### Corsi block tapping task (Kessels et al., 2000)

To measure potential cognitive changes caused by the video gaming intervention we administered a version of the Corsi block tapping task to capture visual spatial working memory capacity. The task consists of the display of nine blue blocks from which at first two are successively highlighted in yellow and the participant is prompted to click on these locations shortly after they were highlighted. Then the number of highlighted blocks is increased in steps of one after two trials. The dependent variable extracted here is how many blocks were successfully remembered in sequence.

#### Digit symbol substitution task (DSST, Thorndike, 1919)

We also administered the DSST to capture processing speed. In this task participants see a table with a mapping between nine symbols and the digits 1–9. The participants are given 2 min time to fill in the respective numbers that correspond to the symbols in a large list of symbols. The dependent variable is how many symbols are successfully associated with the respective number.

#### Manikin test of spatial orientation and transformation (Englund et al., 1987)

The Manikin test consists of the display of a figure who has a green circle and a red square in one of his hands. The figure can face the participant or be displayed showing his back to the participant. Furthermore the figure can be displayed upright or on his head (180° tilted). The figure is surrounded by a green circle or a red square in the background. The task of the participant is to press the left or right button depending on the hand in which the figure holds the colored shape that is displayed in the background.

#### Spatial reasoning task (Salthouse, 2006)

The spatial reasoning task consists of a display of a 2-D folding template of a 3-D shape. The participants have to choose the correct 3-D shape from four different shapes. The participants were given 5 min time and we counted the number of correct answers in this time frame.

#### Trail making test (TMT, Reitan, 1958)

The TMT assesses processing speed and executive functioning. Participants were asked to connect 25 circles as fast as possible, but still being as accurate as possible. In version A, the targets were all numbers (1, 2, 3, etc.) and the participant needed to connect them in sequential order. In version B, the points alternated between numbers and letters (1, A, 2, B, etc.).

All cognitive tests were administered using Inquisit 4 software (http://www.millisecond.com) and we used the tasks as provided online in the Test Library of Inquisit. Completion of the cognitive battery took about 30 min in total. All tasks were performed online on the personal computer of the participants at home. Not all participants took part in this part of the pre- and posttest (*n* = 19), some because they were not able to access the tests for technical reasons, some because they did not respond to the link (see Figure 1).

### Action video game “*Boson X*”

**Figure 2:**
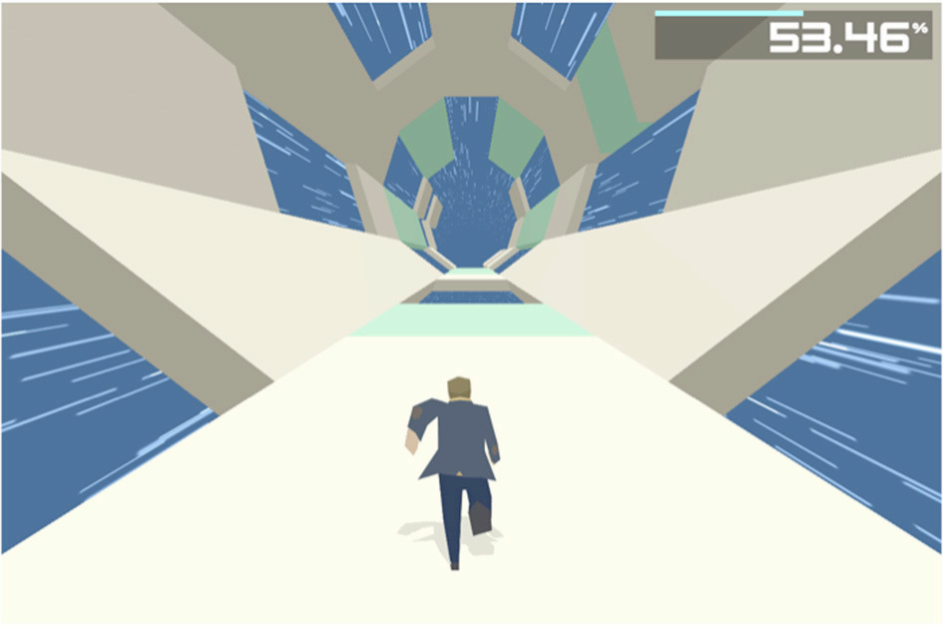
Screenshot taken from the game Boson X.

We selected the action video game *Boson X* (http://www.boson-x.com/) because it can be easily accessed and played on a home computer. In addition, the controls are relatively simple, and can therefore be played by individuals who do not regularly play video games. We collaborated with the game designer Ian MacLarty to obtain a version that enabled us to track game performance parameters. Participants were provided with a link to download the game. *Boson X* is a fast-paced action game in which the player navigates a running avatar (shown from a 3rd person perspective) through a tunnel. The avatar is navigated by pressing the arrow keys on the computer keyboard (arrow pointing upwards = avatar jumps straight ahead, arrow pointing left = avatar jumps forwards to the left, arrow pointing right = avatar jump forwards to the right). The tunnel consists of various tiles and the player has to avoid letting the avatar fall out of the tunnel. If the player steps on a missing tile, the avatar has to repeat the current level. If the player steps on a normal tile, the avatar successfully continues through the tunnel. If the player navigates the avatar onto highlighted tiles, the avatar collects “energy” (Figure 2). The energy status is displayed in the upper right corner of the screen and once 100% energy level is reached the player proceeds to the next level. The more energy is collected the faster the avatar runs and the faster the player needs to respond in order to prevent the avatar from falling out of the tunnel. The slow avatar speed in the early levels of the game, and the fact that responses are limited to only three keys on a normal keyboard, make this game accessible to individuals who do not regularly play video games. As the game progresses and the levels increase, the game demands faster responses and more precise timing. Like most commercial video games, Boson X offers no explicit instructions. However in the piloting phase, individuals who had no prior experience with video games quickly understood what they were supposed to do in the game. Nevertheless, we provided participants with a short manual introducing how to download and install the game, as well as a short description of response buttons and the goal of the game.

### Data analysis

In order to ensure that missingness in the cognitive data and the questionnaire data was completely at random we used the Little missing completely at random (MCAR) test (Little, 1988). Since the test was clearly not significant with $\chi_{\left(4\right)}^{2}$ = 4.014, *p* = 0.404, we decided for listwise deletion of the participants with missingness in the variables of interest so that our final sample for the main analysis consisted of *n* = 29 in the control group and *n* = 21 in the video gaming group.

We employed separate ANCOVAs in which we compared the experimental group with the control group on the dependent variables: depression (BDI, PHQ-9), rumination, subjective cognition, and the cognitive test scores at posttest while controlling for the pretest scores of the respective dependent variable at posttest and habitual video game playing by entering these variables as covariates in the ANCOVA. Sex was entered as a fixed factor, since the sex distribution differed between groups, but most importantly because females are known to play less video games (Lucas and Sherry, 2004), which may reduce the effect on depressivity and cognitive performance.

In separate analyses we correlated the subjective cognition score with TMT performance using a Pearson correlation coefficient. In a post-hoc test we repeated the ANCOVAs for subscores of the subjective cognition score that capture different cognitive domains. We regard effects below a *p*-value of 0.05 as significant. We report η_*p*_^2^ (partial eta square) values as an effect size measure to facilitate the use of it in power analyses to determine the desired sample size for future experiments.

## Results

Patients' demographic characteristics and clinical parameters at pretest are reported in Table 1. The only significant difference at pretest was the distribution of sex (χ^2^ = 0.69, *p* = 0.012) and on the PHQ-9 score [*t*_(48)_ = 2.41, *p* = 0.020], which was higher in the control group. Moreover, we found a trend for BDI score differences at pretest with the same direction [*t*_(48)_ = 2.01, *p* = 0.050] and a tendency for more habitual video game playing in the video gaming group [*t*_(48)_ = 1.99, *p* = 0.052]. Participants in the video game group on average reported to have played two to three times a week (mean = 3.77, *SD* = 2.1). However, due to the fact that participants were randomly assigned to the groups we assume that the differences in sex and PHQ-9 arose by chance and are not systematically related to the dependent variable which makes it feasible to use ANCOVA as a statistical procedure.

**Table 1:** Pretest characteristics of the sample that completed the study.

	**Video game group (*n* = 21)**	**Control group (*n* = 29)**	**Statistics**
Age	45.0 (11.2)	45.3 (12.0)	*t*_(48)_ = 0.083, *p* = 0.935
Sex (male/female)	10/11	4/25	χ^2^ = 0.69, *p* = 0.012^*^
School	6.43 (0.87)	6.45 (0.87)	*t*_(48)_ = 0.079, *p* = 0.937
Duration of illness (in years)	12.68 (2.8)	12.76 (2.4)	*t*_(45)_ = −0.69, *p* = 0.492
Currently in treatment (no/yes)	12/8	20/7	χ^2^ = 0.31, *p* = 0.355
Antidepressant medication (no/yes)	12/9	15/14	χ^2^ = 0.70, *p* = 0.778
Antipsychotic medication (no/yes)	21/0	26/3	χ^2^ = 2.31, *p* = 0.128
Sedative medication (no/yes)	20/1	27/2	χ^2^ = 0.10, *p* = 0.754
Number of psychiatric comorbidities	0.80 (0.17)	0.92 (0.17)	*t*_(48)_ = 1.24, *p* = 0.22
PHQ-9	8.33 (3.4)	11.45 (5.2)	*t*_(48)_ = 2.41, *p* = 0.020^*^
BDI	14.76 (7.1)	19.86 (9.9)	*t*_(48)_ = 2.01, *p* = 0.050
RSQ	21.42 (4.7)	22.24 (5.3)	*t*_(48)_ = 0.56, *p* = 0.576
SSTICS	77.19 (13.6)	78.41 (14.4)	*t*_(48)_ = 0.30, *p* = 0.763
SSTICS WM	3.0 (1.0)	2.9 (1.2)	*t*_(48)_ = −0.55, *p* = 0.505
SSTICS EM	3.9 (0.6)	4.0 (0.7)	*t*_(48)_ = 0.36, *p* = 0.721
SSTICS SM	3.5 (0.8)	3.8 (0.8)	*t*_(48)_ = 1.22, *p* = 0.230
SSTICS Att	3.3 (0.9)	3.4 (0.9)	*t*_(48)_ = 0.43, *p* = 0.672
SSTICS Exec	4.0 (0.8)	3.9 (1.0)	*t*_(48)_ = −0.39, *p* = 0.702
Habitual video game playing (in hours per week)	12.03 (15.2)	5.31 (8.6)	*t*_(48)_ = −1.99, *p* = 0.052

*Means, standard deviations (in brackets) and frequency. PHQ-9, Patient Health Questionnaire-9; BDI, Beck Depression Inventory; RSQ, Response Styles Questionnaire; SSTICS, Subjective Scale to Investigate Cognition in Schizophrenia; WM, working memory; EM, episodic memory; SM, semantic memory; Att, attention; Exec, executive functioning*.

Our main focus was on potential changes in depressive symptoms but also on associated symptoms such as rumination and subjective cognition due to our video game intervention. In separate ANCOVAs we compared the video game group against the passive control group at posttest and found a significant effect of group reflecting lower rumination score in the video gaming group [*F*_(1, 44)_ = 72.65, *p* = 0.017, η_*p*_^2^ = 0.123, with the covariate of pretest rumination score being significant, *p* < 0.001, see Table 2], as well as significantly higher subjective cognition in the video gaming group at posttest [*F*_(1, 44)_ = 162.31, *p* = 0.043, η_*p*_^2^ = 0.090, with the covariate of pretest subjective cognition score, *p* < 0.001] and habitual video gaming (*p* = 0.035) being significant. For the same analysis on BDI scores [*F*_(1, 44)_ = 1.67, *p* = 0.167, η_*p*_^2^ = 0.043, with the covariate of pretest BDI score being significant, *p* < 0.001] and PHQ-9 scores [*F*_(1, 44)_ = 0.503, *p* = 0.482, η_*p*_^2^ = 0.011, with the covariate of pretest PHQ-9 score, *p* < 0.001] we did not find a significant group effects at posttest. When correcting for multiple testing by means of Bonferroni correction (three more or less independent tests with an overall *p*-value criterion of *p* < 0.05 results in a corrected *p*-value of 0.0125) none of the analyses presented would survive this correction. The changes from pre- to posttest in subjective cognition, BDI, PHQ9 and rumination were not associated with self-reported playtime at posttest (*p* > 0.354).

**Table 2:** Pre- and posttest symptom questionnaires of the sample that completed the study.

	**Video game group (*****n*** = **21)**		**Control group (*****n*** = **29)**		**Statistical results**		
	**Pretest**	**Posttest**	**Pretest**	**Posttest**	**Pretest differences**	**ANCOVA results**	**Sig. covariates**
PHQ-9	8.33 (3.4)	7.81 (3.5)	11.45 (5.2)	10.45 (5.2)	*t*_(48)_ = 2.41, *p* = 0.020^*^	*F*_(1, 44)_ = 0.503, *p* = 0.482	PHQ-9 pretest
BDI	14.76 (7.1)	11.76 (7.4)	19.86 (9.9)	17.72 (9.5)	*t*_(48)_ = 2.01, *p* = 0.050	*F*_(1, 44)_ = 1.67, *p* = 0.167	BDI pretest
RSQ	21.42 (4.7)	19.10 (4.4)	22.24 (5.3)	21.83 (4.9)	*t*_(48)_ = 0.56, *p* = 0.576	*F*_(1, 44)_ = 72.65, *p* = 0.017^*^	RSQ pretest
SSTICS	77.19 (13.6)	81.14 (11.7)	78.41 (14.4)	79.03 (14.0)	*t*_(48)_ = 0.30, *p* = 0.763	*F*_(1, 44)_ = 162.31, *p* = 0.043^*^	SSTICS pretest, gaming
SSTICS WM	3.0 (1.0)	3.4 (0.9)	2.9 (1.2)	3.1 (1.0)	*t*_(48)_ = −0.55, *p* = 0.505	*F*_(1, 44)_ = 0.718, *p* = 0.401	SSTICS WM pretest
SSTICS EM	3.9 (0.6)	4.1 (0.5)	4.0 (0.7)	4.0 (0.6)	*t*_(48)_ = 0.36, *p* = 0.721	*F*_(1, 44)_ = 4.691, *p* = 0.036^*^	SSTICS EM pretest
SSTICS SM	3.5 (0.8)	3.9 (0.6)	3.8 (0.8)	3.7 (0.8)	*t*_(48)_ = 1.22, *p* = 0.230	*F*_(1, 44)_ = 3.368, *p* = 0.073	SSTICS SM pretest
SSTICS Att	3.3 (0.9)	3.3 (0.8)	3.4 (0.9)	3.3 (1.0)	*t*_(48)_ = 0.43, *p* = 0.672	*F*_(1, 44)_ = 0.295, *p* = 0.590	SSTICS Att pretest
SSTICS Exec	4.0 (0.8)	4.2 (0.9)	3.9 (1.0)	4.1 (0.9)	*t*_(48)_ = −0.39, *p* = 0.702	*F*_(1, 44)_ = 0.903, *p* = 0.347	SSTICS Exec pretest

*Means, standard deviations (in brackets). PHQ-9, Patient Health Questionnaire-9; BDI, Beck Depression Inventory; RSQ, Response Styles Questionnaire; SSTICS, Subjective Scale to Investigate Cognition in Schizophrenia; WM, working memory; EM, episodic memory; SM, semantic memory; Att, attention; Exec, executive functioning*.

* *p < 0.05*.

When exploring the subsample with data on cognitive performance (*n* = 10 control group, *n* = 9 video game training group, Table 3) we found a significant posttest group difference in performance on TMT performance when controlling for habitual video game playing, pretest TMT performance and accounting for sex, with the video game group showing faster performance compared with the control group [*F*_(1, 19)_ = 5.06, *p* = 0.030, η_*p*_^2^ = 0.103, the covariate of pretest performance, *p* = 0.034, was significant and sex was close to significance, *p* = 0.052]. We found no significant effects in the performance data of the other cognitive tests (*p* > 0.261). The TMT result does not survive when applying Bonferroni correction to adjust for multiple testing. TMT performance data was uncorrelated to the subjective cognition score at pretest [*r*_(19)_ = −0.026, *p* = 0.916] as well as at posttest [*r*_(19)_ = −0.273, *p* = 0.258].

**Table 3:** Pre- and posttest performance in the cognitive test sample.

	**Video game group (*****n*** = **9)**		**Control group (*****n*** = **10)**	
	**Pretest**	**Posttest**	**Pretest**	**Posttest**
Trail making task A and B (in sec)	142 (92)	119 (46)	158 (44)	131 (29)
Corsi block tapping (sum correct)	49 (24)	52 (24)	50 (17)	51 (16)
Digit symbol (sum correct)	49 (24)	58 (36)	48 (11)	47 (9)
Manikin (sum correct)	41 (10)	41 (8)	43 (8)	43 (8)
Spatial relations (sum correct)	9 (4)	9 (5)	10 (2)	11(4)

*Means, standard deviations (in brackets)*.

To explore the finding of an increase in subjective cognition in the experimental training group in more detail, we decomposed the SSTICS total score according to the cognitive domains that the items of the SSTICS referred to. While controlling for habitual video game playing and the pretest score and accounting for sex, as described above, we found that the subscore of episodic memory was significantly higher [*F*_(1, 44)_ = 4.691, *p* = 0.036, η_*p*_^2^ = 0.096], and the semantic memory showing a tendency for higher values in the experimental group at posttest [*F*_(1, 44)_ = 3.368, *p* = 0.073, η_*p*_^2^ = 0.071].

## Discussion

Within the scope of the present training study we found results suggesting that a 6-week training with a fast paced action video game (Boson X) in depressed patients contributes to lower levels of rumination and higher levels of subjective cognitive ability at posttest in comparison to a control group (although these results did not survive multiple comparison correction). On a small subsample, we detected better processing speed and cognitive flexibility (TMT) in the training group at posttest compared with a passive control group when controlling for pretest performance, pointing into the direction of our hypothesis that fast paced action video games may train executive functioning.

Although we did not find effects on the measure of depression severity (BDI, PHQ-9), the observed reduction in rumination is highly relevant with respect to depression. The measure we used to assess rumination is based on the response style theory (Nolen-Hoeksema, 1987) according to which rumination is characterized by self-reflection and a passive focus on negative emotions. Rumination can exacerbate negative moods, and can both trigger and prolong depressive episodes (Nolen-Hoeksema et al., 2008).

Taken together our results are an interesting add on to findings of Russoniello and colleagues, who trained depressed patients with casual puzzle-like video games (Russoniello et al., 2013). But differently to Russoniello's finding of a reduction in PHQ-9 scale, our data suggest a reduction of rumination. The difference in outcome between this and our study might be due to the game genre used in the intervention. The previous literature on action video games suggests improvements on executive functions (Powers et al., 2013). Executive functions comprise the ability to inhibit ongoing actions as well as thoughts. Rumination in turn has been associated with deficits in brain areas associated with inhibition (Kühn et al., 2012, 2014b; Yang et al., 2017). Action video games may improve the ability to supress rumination via improvements in executive function, but less effectively target the affective symptoms of depression. In contrast, puzzle-like video games might be more rewarding and less frustrating, and therefore better suited to entertain patients and therewith cause changes in mood. Unfortunately, our cognitive battery did not include any inhibition tasks to explore this hypothesis further. However, this is a post-hoc speculation that may inspire future research. Interestingly, a previous study that applied a working memory training regime targeted at rumination to depressed patients failed to show a reduction in rumination (Wanmaker et al., 2015). This finding underlines the recently emerging notion that cognitive training regimes may train certain processes but often show little transfer effects to other non-trained domains (Melby-Lervag et al., 2016). One may interpret the finding as in line with the fact that we do observe a reduction of rumination, but no effect on our spatial working memory task (Corsi block tapping).

In addition to the observed reduction in rumination we found an effect on the subjective assessment of cognitive functioning, albeit neither of the effects survive Bonferroni correction. Although the latter subjective cognition scale we used was originally used in schizophrenia patients, the questions are not disease specific and the same cognitive domains (working memory, episodic memory, semantic memory, attention, executive functioning) are also highly relevant in depression. Although the size of the subsample from which we obtained cognitive data was admittedly fairly small, we did not observe an association between objective performance and subjective assessment of cognition. This is in line with previous research on depressed patients failing to show correlations between objective and subjective cognitive performance data (Srisurapanont et al., 2017). The authors demonstrated that age and depression severity may be predictive of the discrepancy between subjective and objective measures. In a study on a large healthy population cohort of whom 87% reported at least one symptom of depression, likewise no association between subjective memory complaints and objective memory performance was shown, although participants showed a decline in memory for pictures presented in a negative context (Schweizer et al., 2017).

Interestingly, in an additional analysis in which we set out to explore which cognitive domain contributed most to the overall effect of an increase in subjective cognition, we computed separate analyses for each domain and found that in particular episodic memory performance subjectively increased in response to the video game intervention. Unfortunately, we did not assess any neuropsychological tasks that tap into the episodic memory domain in the present study.

In the subsample from which we obtained cognitive performance data, we found a reduction in reaction time in the TMT, which is a measure of executive functioning. However, we did not find any effects on the spatial working memory task (Corsi block tapping task), the speed of processing task (DSST) or the two spatial processing tasks (Manikin task, Spatial relations task). The absence of an effect on processing speed is in line with a recent study on healthy adults who have been trained with an action video game that likewise found no improvement in a speed task (van Ravenzwaaij et al., 2014). However, a previously published study did show effects of action video gaming on speed of processing (Dye et al., 2009) and since Trail Making, for which we found a significant improvement in the present study, also captures speed of processing to a certain extent one may have expected to observe improvement in the Digit symbol substitution task as well.

We selected three spatial tasks (Corsi block tapping, Manikin task, Spatial relations task) based on the reasoning that the spatial nature of the tasks may be a proxy of hippocampal functioning and previous studies have demonstrated positive effects of action video gaming on spatial processing. In one study, pre-existing gender differences in spatial processing were eliminated after 10 h of action video game training and in another study, training with action video games was demonstrated to improve performance on a virtual surgery task in medical students (Feng et al., 2007; Schlickum et al., 2009). Since depression has repeatedly been associated with volume decrements in the hippocampal formation, and antidepressant use with neurogenesis in the hippocampus (Jacobs et al., 2000; Willner et al., 2013) we reasoned that spatial tests could be an indirect measure of brain structural changes known to be elicited by action video games (Kühn et al., 2014a).

## Limitations

A profound limitation of the study is the overall sample size, as well as the small sample size in the subgroup that completed the neuropsychological test battery before and after the intervention. This may have considerably reduced the power of the present design to detect effects.

Another critical point is that the observed effects, in particular on self-reported subjective cognition, may have been caused by the non-blind nature of the intervention and may reflect the participants' expectation that the game improved their cognition. However, at least in Germany, where the study was performed, video games are not very highly valued, which may also have lead participants to believe that video games should impair their cognition, although this was not assessed. Future work may usefully explore how factors such as expectation and motivation may influence the efficacy of video game interventions. Another limitation is that we were not able to evaluate potential associations between performance in the video game to the outcome, since we did not obtain the game logging information from all participants. Future studies should carefully log performance data as it may reveal insights into the particular elements of the game that drive the observed effects. Furthermore, the fact that the testing and training was accomplished by the participants at their home computer is a disadvantage because it is not well-controlled how much participants trained and whether participants engaged in other activities while filling in questionnaires or during the training. Future studies should likewise control the training time to investigate dose-response relationships.

Due to the fact that the control group in the present study did not perform any task, we cannot finally exclude that the observed effects are due to distraction in the experimental group that was absent in the control group. However, this is unlikely to be the only mechanism at work here, as we observed the predicted effects of action video games on executive function, similar to previous work in healthy individuals (Green and Bavelier, 2003; Dye et al., 2009; Colzato et al., 2013). Nevertheless, rather than a passive control group, future studies should use an active control group that gets an instruction to pursue a certain activity e.g., reading books on a tablet to control for differences in motivation and to exclude the possibility that the observed effects are purely due to distraction. As our questionnaires retrospectively ask about a time frame of 1–2 weeks, responses may have differed depending on whether participants were still engaged in the game or not. To prevent this, future studies should consider administering the posttest questionnaires 2 weeks after the end of the training interval.

Taken together, the present results suggest beneficial effects of training with an action video game on executive function, rumination and subjective cognition in a sample of depressed patients, albeit the results do not survive multiple comparison correction. These results pave the way for future studies that may set out to unravel the potentially mediating role of cognitive changes in the beneficial effects of action video game training on rumination in depressed patients. We did not observe significant improvements in affective symptoms following an action video game intervention. Other games, such as puzzle-like video games, might be better suited to improve mood in depression (Russoniello et al., 2013). Future work on video game interventions for depression may seek to combine both action and puzzle games, to target affective and cognitive symptoms simultaneously. A clear advantage of video game interventions is that they constitute a low-cost, feasible intervention that can be easily offered to patients alongside regular therapy, and even as an online offer to extend treatment to their home computer. Administration does not require a clinician, and can even be self-administered. Video games are enjoyable to play, and, unlike pharmacological interventions for depression, have not been found to produce adverse side effects so compliance is likely to be high. The games are also adaptive; as an individual continues to play the difficulty level (e.g., the speed at which the avatar moves) automatically increases until the game ends (the avatar falls out of the tunnel), at which point the game restarts. As such, through adaptation of the level of difficulty to the skill of the player, frustration that the game is too difficult and boredom that the game is too easy should be kept at a minimum and the player should remain engaged in the game. As such, video games provide a promising, novel, and highly practical therapeutic intervention for individuals with depression.

## Author contributions

Contribution to the design of the study: SK, SM, and JG; to the acquisition of the data: SK, SM, FB, and TL; to data analysis: SK, SM, and FB; to drafting the manuscript: SK, SM, FB, TL, and JG. All authors take full responsibility for the content of the paper.

### Conflict of interest statement

The authors declare that the research was conducted in the absence of any commercial or financial relationships that could be construed as a potential conflict of interest.
